# Major vessel sealing in laparoscopic surgery for colorectal cancer: a single-center experience with 759 patients

**DOI:** 10.1186/s12957-018-1402-x

**Published:** 2018-06-01

**Authors:** Michele Grieco, Daniela Apa, Domenico Spoletini, Emanuela Grattarola, Massimo Carlini

**Affiliations:** 1 General Surgery Department, S. Eugenio Hospital, P.le dell’Umanesimo 10, 00144 Rome, Italy; 2 Statistical and Big Data Department, Elis Consulting and Labs, Via S. Sandri 81, 00159 Rome, Italy

**Keywords:** Colon cancer, Laparoscopic surgery, Vessel sealing

## Abstract

**Background:**

Efficient hemostatic techniques are essential in laparoscopic surgery for ideal intraoperative and postoperative results. A variety of advanced devices are available for the sealing of major vascular structures. The aim of this study is to assess effectiveness and safety of major vessel sealing with a radiofrequency device during laparoscopic colorectal resections for cancer based on the experience of a single hospital.

**Methods:**

Early outcomes of a consecutive series of patients who received elective laparoscopic colorectal resections for cancer over a 10-year period (January 2008–September 2017) are analyzed.

In all procedures, the Ligasure® electrothermal bipolar device was used for the closure of the major colonic vessels and the dissection of all the structures. No other products such clips, staplers, hemostatic products, or other devices were used.

**Results:**

Seven-hundred fifty-nine procedures were performed in laparoscopy: 179 rectal resections, 247 sigmoidectomies and left hemicolectomies, 240 right hemicolectomies, 33 resections of the splenic flexure, 35 transverse colonic resections, and 25 other procedures.

In 39 cases, the laparoscopic procedure was converted to open surgery, and in these cases, vessel sealing was also achieved with the radiofrequency device alone.

Vessel dissection and sealing was realized in all cases without any intraoperative or postoperative bleeding. No reoperations for bleeding from major vessels were performed in any patients. One case of reoperation was recorded postoperatively, at 3 h after right hemicolectomy, due to a small bleeding from the fat of the transverse colon stump.

**Conclusions:**

The use of Ligasure® radiofrequency device for sealing and dividing the major colonic vessels is safe, fast, and effective during laparoscopic colorectal resections.

## Introduction

Laparoscopic colorectal resection has become widespread due to the well-known benefits that it provides. Simultaneously, the use of energy-based devices is increasing worldwide alongside with decreases in the use of electrical monopolar and bipolar devices.

Conventional monopolar tools increase the risk of thermal injury, make hemostasis difficult, produce smoke, and often require additional tools, such as bipolar graspers, sutures, and clips, to achieve efficient hemostasis and vessel sealing.

Currently, new energy-based devices are available to reduce the length of surgery and minimize blood loss while producing a major hemostatic effect. Most of these devices use radiofrequency (i.e., electrothermal bipolar vessel sealers; EBVSs), ultrasound (i.e., ultrasonic shears; USs), or even combine these energies with conventional bipolar energy [1].

The main advantage of these modern technologies is enhanced sealing capability, especially in blood vessels larger than 2–4 mm in diameter [2]. These technologies are particularly efficient for the sealing of large vessels of up to 5–7 mm in diameter through compression and efficient energy delivery to the tissue [3].

Radiofrequency (Ligasure®) and ultrasound (Ultracision®) devices are the most popular in general surgery [4]. Surgical hemostasis with EBVSs is accomplished by delivering high-frequency and low-voltage electricity that is converted into thermal energy. This technology is able to measure the impedance of the tissue between the jaws and adapt the electrical power to obtain the most efficient final sealing. The energy, combined with the compression of the jaws, collapses the vessel walls and denatures the collagen and elastin to form rearranged tissue that provides a hemostatic seal [5, 6].

In this study, experiences of major vessel sealing during laparoscopic surgeries for colorectal cancer using the Ligasure® radiofrequency device are reported. It is a very large series of a single center that support the safety and effectiveness of EBVS for vessel sealing.

## Methods

### Patient selection

In this retrospective, observational, clinical study, the early outcomes of colorectal laparoscopic resections in 759 unselected consecutive cases during operations at the S. Eugenio Hospital (Rome, Italy) over a 10-year period from January 2008 to September 2017 are reported. The major colonic vessels were closed and divided with a 5-mm Ligasure®.

Eligible patients included unselected adults who underwent elective laparoscopic colorectal resection for cancer for whom the dissection, sealing, and sectioning of major colorectal vessels were obtained with the exclusive use of the Ligasure® radiofrequency device. Patients who were converted to open surgery were included if no other devices were used to seal the major vessels.

The exclusion criteria were the use of other devices for the vessel sealing. In all cases, the radiofrequency device was used in every step of the procedure.

### Surgical technique

In the right hemicolectomies, the ileocolic vessels and the right branch of the middle colic artery were sealed. The middle colic vessels were closed and divided in transverse colectomies. The left branch of the middle colic vessels and the left colic artery were sealed and dissected during resections of the splenic flexure. The inferior mesenteric artery was sealed and dissected during left colectomies, sigmoidectomies, and rectal resections.

The major arterial branches were closed and cut after five consecutive activations of the device using bipolar energy alone for the first four applications, and additional cutting was performed at the end of the final application. The first four applications were performed along the vessel, and each activation overlapped with the previous activation. The final application and cutting were performed in the middle of the sealed artery.

The major venous branches were sealed after three activations while leaving a thin layer of fatty tissue around the structures to reinforce the sealed tissue line with the rearrangement of the components of the connective tissue. Electrothermal energy alone was applied during the first two consecutive applications, and the cutting was performed at the end of the third application in the middle of the sealed vein.

The device was applied perpendicularly to the vessel to minimize the length of the seal and to improve the seal strength [7]. The dissections of all the non-vascular structures (i.e., the mesocolic root, ligaments, peritoneum, and omentum) were performed with single applications.

### Data collection

Each type of vessel closure, the time and the number of applications were assessed. The use of additional hemostatic products was noted. The type of colectomy, surgery time, intraoperative and postoperative bleeding, length of hospital stay, and medical and surgical morbidities and mortalities were recorded.

Post-operative morbidity was defined and classified according to the Clavien-Dindo Classification [8].

### Outcomes

The primary outcomes were major post-operative bleeding and reoperations for bleeding. The secondary outcomes were the overall 30-day morbidity and mortality.

### Statistical analysis

Quantitative data are reported as the mean ± the standard deviation (range). Qualitative data are reported as the numbers of patients (percentages of patients).

All analyses were performed using SPSS software version 23 (IBM Co., Armonk, NY, USA).

## Results

### Study population

The population was composed of 759 consecutive unselected patients (339 males and 420 females) The mean age was 68.2 ± 10.92 (22–89) years, the mean BMI was 26.0 ± 3.77 (18.7–35.9) kg/m^2^, the rate of previous intraperitoneal surgery (laparoscopic or laparotomic and major or minor) was 30.8%, 150 patients were ASA I, 419 patients were ASA II, and 190 patients were ASA III (Table 1).

**Table 1:** Demographic features

Patients (*n*)	759
Gender (*n*, %)	
Male	339 (44.7)
Female	420 (55.3)
Age, years (mean ± SD, range)	68.2 ± 10.92 (22–89)
BMI, kg/m2 (mean ± SD, range)	26.0 ± 3.77 (18.7–35.9)
ASA Classification (*n*, %)	
I	150 (19.8)
II	419 (55.2)
III	190 (25.0)
Previous abdominal surgery (*n*, %)	234 (30.8)

The operative procedures included the following: 240 right hemicolectomies, 179 rectal resections (105 proximal rectums, 74 middle and distal rectums), 180 sigmoidectomies, 67 left hemicolectomies, 35 transverse colonic resections, 33 resections of the splenic flexure, 5 abdomino-perineal amputations, 10 Hartmann procedures, 3 total colectomies, and 7 multiple resections (Table 2).

**Table 2:** Procedures

Right hemicolectomy	240
Transverse colon resection	35
Splenic flexure resection	33
Left hemicolectomy	67
Sigmoidectomy	180
Rectal resection with PME	105
Low rectal resection with TME	74
Hartmann procedure	10
Miles procedure	5
Multiple resection	7
Complete colectomy	3
Total	759

The number of device applications used to seal the ileocolic, middle colic, and inferior mesenteric arteries was 5. No thermal injuries to other structures were recorded. Moreover, no other energy devices, clips, staplers, or additional hemostatic products were necessary to achieve hemostasis. The average time required to seal and transect the arteries was 25 ± 3.77 (20–32) s.

For the veins, complete hemostasis was also obtained in all cases. No further hemostatic products or additional devices were required. Usually, the inferior mesenteric vein was skeletonized while leaving a thin layer of fatty tissue surrounding it to reinforce the sealed tissue line with the rearrangement of the components of the connective tissue. The average major vein transection time was 15 ± 2.77 (10–18) s.

### Outcomes

Vessel sealing was effective in 100% of cases without any intraoperative or postoperative bleeding from the major colonic vessels. All of the procedures began with laparoscopy, and 39 procedures (5.1%) were converted to open surgery. In these cases, vessel sealing was also achieved with the radiofrequency device alone.

In all of the procedures, tissue dissection was achieved with no thermal injuries to the surrounding structures. The mean blood loss was 37.5 ± 18.4 (10–300) ml.

Only one reintervention for hemostasis was performed at 3 h after a right hemicolectomy for venous bleeding from the pericolic fat of the transverse stump due to an epiploic fringe that was grabbed in the staple line. The reoperation was performed with a minimally invasive approach.

The mean operative time was 123.0 ± 36.3 (65–240) min. Regarding postoperative morbidity, Clavien-Dindo scores of II, III, or IV occurred in 161 patients (rate 21.2%). Anastomotic leaks occurred in 30 patients (4%). Nine perioperative deaths (1.2%) were recorded (Table 3).

**Table 3:** Outcomes

Operative time, min (mean ± SD, range)	123.0 ± 36.3 (65–240)
Conversions (*n*, %)	39 (5.1%)
Estimated intraoperative blood loss, ml (mean ± SD, range)	37.5 ± 18.4 (10–300)
Intraoperative hemorrhaging (*n*, %)	0 (0%)
Early reoperation for minor bleeding (*n*, %)	1 (0.1%)
Morbidity: Clavien-Dindo score ≥ 2 (*n*, %)	161 (21.2%)
Anastomotic leak (*n*, %)	30 (4%)
Mortality (*n*, %)	9 (1.2%)

## Discussion

In this clinical study, the Ligasure® device was successfully used to divide and seal the ileocolic, middle colic, and inferior mesenteric vessels in a variety of laparoscopic colorectal procedures.

In this series, three or five activations of the device were sufficient to provide and maintain adequate hemostasis of the major veins and arteries in all cases.

The exposure of the colorectal vessels varied among the patients and procedures, but this did not appear to be a critical factor in vessel sealing. Although the capacity of the device to seal large vessels is undoubted, the veins were prepared leaving a thin layer of fatty tissue around them to achieve more fibers that were sealed on the transection line and to ensure a safe seal [5, 6].

To optimize the performance of the device, verification of the appropriate size of the vessel and its complete inclusion in the jaws of the device is suggested. The release of the tension on the vessel during sealing is recommended to allow for the completion of the sealing cycle before cutting.

Essential factors mandatory for blood vessel sealing include homogeneously distribution of compression and precise temperatures and times to permit for the optimal formation of strong sealed vascular tissue. Perpendicular closure and uniform tissue compression of the jaws minimizes the length of the sealed tissue and improves its strength for very consistent sealing [7].

During laparoscopic procedures, due to the wide and blunt jaws of the radiofrequency device, it can be used as a non-traumatic grasping instrument. This feature provides the surgeon the possibility of maintaining the same surgical device in his right hand throughout the procedure until the final step of stapling and sectioning the bowel.

The availability of such a multifunctional device may also be of particular value in complex laparoscopic procedures, such as colorectal resections, because it reduces the number of devices necessary to achieve safe and reliable hemostasis and avoids the continuous changing of tools. The avoidance of multiple instruments is a time- and cost-saving strategy.

Radiofrequency devices are often compared with two other main categories, ultrasonic instruments and advanced bipolar systems. The effects of sealing of these two technologies are limited compared with radiofrequency devices; however, both devices can efficiently seal structures as vessels (≤ 5 and ≤ 7 mm of vessel diameter, respectively).

All these technologies are the main topic of few comparative studies, to confront their relative advantages and disadvantages. Currently, there is no consensus on the superiority of a device above the other [4, 9, 10].

In a review study of the Cochrane Database, Tou S [9] analyzed six randomized controlled trials that included 446 participants. All of the patients underwent elective laparoscopic or laparoscopic-assisted right, left or total colectomies or anterior resections for either benign or malignant diseases.

Few trials have compared three types of instruments, i.e., monopolar electrocautery shears (MES), ultrasonic shears (US), and EBVS. Significantly less blood loss has been observed with US compared to MES (MD 42 ml, 95% CI 22–62) and no significant difference comparing EBVS with US or MES. In terms of operating, time is significantly shorter with the use of EBVS than MES (MD 40 min, 95% CI 17–63) and shorter but not significant between US and MES, no differences between US and EBVS have been observed. Hemostatic control with US and EBVS is better than that of MES. The authors state an overall better hemostasis and shorter operating time of US and EBVS over MES, but there was no difference in outcomes between these two instruments. EBVS appeared just to be easier to handle than US.

No conclusions regarding cost differences between these three instruments have been noted.

In 2012, Di Lorenzo et al. [11] published a meta-analysis that included four studies comparing EBVS and US in 397 patients (200 EBVS vs. 197 US patients). The findings indicated that EBVS is associated with a significantly shorter operative time and less intraoperative blood loss than US (*p* < 0.05) in laparoscopic colorectal surgery. However, these results should be interpreted with caution due to the high heterogeneity of the trials and the limited number of studies with a high level of evidence.

Janssen et al. [12] compared 7 RCTs in a systematic review, including 554 patients in total. The use of US (*n* = 139) were compared to those that used of EBVS (*n* = 264), monopolar (*n* = 20) or bipolar devices (*n* = 130). Two studies were in favor of EBVS with shorter operating time, reduced intraoperative blood loss, and lower cost. However, in the other studies no differences were observed.

The authors confirm that, vessel sealing with advanced devices such EBVS and US may be considered relatively safe, and their use may reduce costs of surgery for the reduction of blood loss and the operating times in many abdominal surgical procedures compared to mono- or bipolar devices.

Martin et al. [13] examined a large retrospective series of 802 cases who underwent elective laparoscopic colorectal cancer resections that were performed with EBVS. Effective vessel sealing was achieved in 99.8% of the cases. Another recent study by Cassini et al. [14] underlined the benefits of radiofrequency devices in terms of lower intraoperative blood loss and shorter operative times in obese patients with colorectal cancer.

Similar results have also been reported for pancreatic, biliary, and lung surgeries [15–17].

The bursting pressures of arteries sealed with US, EBVS, titanium laparoscopic clips (LC), and plastic laparoscopic clips, were measured in an experimental animal study [18]. Arteries were divided in three size groups (2–3, 4–5, and 6–7 mm) and were harvested from freshly euthanized animals. The EBVSs mean burst pressures was significantly higher compared to the US at 4–5 mm and 6–7 mm, with a pressure of 601 vs 205 mmHg and 442 vs 175 mmHg, respectively. The burst pressures of the US and EBVS at 2 or 3 mm were not significantly different.

The EBVS showed a higher burst pressures for the 4–5 mm group and the 6–7 mm group, compared with the 2–3 mm group were the pressure was 128 mmHg (*p* = 0.0001).

Clips were statistically stronger than the two devices in 2–3 mm group and 6–7 mm group, but not in the 4–5 mm group where the EBVS was as strong as the LC (601 vs 593 mmHg).

EBVS was safe and effective in vessels up to 7 mm where can be used confidently. Concerning the thermal spread to the surrounding tissues, no differences were showed between the EBVS and US devices.

In another study by Noble et al. [19], 93 vessels from 18 patients were sealed with Lotus™ (33 patients), Harmonic Ace® (30 patients), and Ligasure® (30 patients). The mean bursting pressures were 1170 for the Lotus™, 1470 for the Harmonic Ace®, and 1510 mmHg for the Ligasure®. Therefore, the bursting pressure seems really high should be considered that the bursting pressures of mesenteric vessels, sealed with the bipolar instruments, are significantly higher than physiological pressures, as also documented in a study by Bibi S [20].

## Conclusions

The results of this large single-center series demonstrates that use of the Ligasure® radiofrequency device to seal and divide the major vessels in laparoscopic colorectal resections is safe, fast and effective, and associated with a very high success rate, as already demonstrated in existing literature. This device reduces the operative time, number of surgical instruments, and costs.

## Abbreviations

ASA: American Society of Anesthesiology Score
EBVS: Electrothermal bipolar vessel sealer
LC: Laparoscopic clip
MES: Monopolar electrocautery shears
US: Ultrasonic shears
